# Expression of TAAs in glioblastoma and expansion of anti-TAA -reactive T cells

**DOI:** 10.1186/2051-1426-2-S3-P24

**Published:** 2014-11-06

**Authors:** Ernest Dodoo, Liu zhenjiang, Bartek Jiri, Oscar Persson, Qingda Meng, Thomas Poiret, Lalit Rane, Christopher Illies, Julia Karbach, Elke Jäger, Markus Maeurer

**Affiliations:** 1 Dept. of Neurosurgery, Karolinska University Hospital, Stockholm, Sweden; 2 Karolinska Insitutet, Stockholm, Sweden; 3 Krankenhaus Nordwest, Frankfurt/M., Germany

## Purpose

Active cellular therapy (ACT) using *ex-vivo *expanded T cells from patients with cancer, obtained by apheresis, can represent a viable source for anti-cancer directed cellular therapy. TAAs expressed in glioblastoma may represent attractive targets for i) CARS, ii) transgenic T cells targeting nominal tumor antigens (e.g. NY-ESO-1) or iii) T cells enriched for TAA after *ex vivo *expansion.

## Methods

Fresh blood samples were obtained from 50 patients with tumors of the central nervous system and tested for anti-TAA reactivity. T cells were expanded without cytokines, with IL-2 and IL-7, or with IL-2, IL-15 and IL-21 and tested for CD4/8 expansion by flow cytometry and for IFN-gamma production by ELISA. PBMCs were expanded using IL-2/15/21 and peptides (15mers) covering TAAs (surviving or NY-ESO-1). The T cell phenotype (CD3, CD4, CD8, CD45RA and CCR7) was determined by flow cytometry and TAA-reactive T cells were identified by intracellular cytokine staining (IL-2, TNF, IFN and IL-17). TAA-specific IgG in serum was detected by a quantitative ELISA in patients with glioblastoma and in age- and sex-matched healthy donors. FGFRvIII was determined by RT-PCR and protein expression of survivin and NY-ESO-1 was evaluated by immunohistochemistry and graded using a scale from 1+ to 4+ along with pattern analysis of TAA expression.

## Results

We could detect IFN-gamma responses in 25% blood samples for NY-ESO-1 and 30% for survivin and antigen-specific CD8 / CD4+ T cell proliferation. Cellular responses could be augmented by adding cytokines, i.e. IL-2 and IL-7 favored CD4+ T cell proliferation, IL-2, IL-15 and IL-21 favored CD8+ T cell proliferation. TAAs-reactive T cells could be successfully expanded ex vivo and exhibited TAA-specific production of IFNgamma and TNFalpha and a CD8+CD45RA-CCR7+ phenotype. 38/50 specimens expressed NY-ESO-1, yet only 3/50 showed a strong, universal (4+) NY-ESO-1 protein expression pattern, 12/50 cancer lesions exhibited a strong (4+) staining for survivin defined by immunohistochemistry. 25 % of glioblastoma tested positive for the FGFRvIII.

## Conclusion

A TAA-specific WBA (whole blood assay) can be used to gauge the potential for expansion of TAA-reactive T cells in peripheral blood from patients with glioblastoma. TAA-reactive T cells can be successfully expanded from patients with glioblastoma in IL-2, IL-15 and IL-21, they exhibit a central memory phenotype and produce a Th1 cytokine cytokine pattern. NY-ESO-1 expression in glioblastoma represents a viable target for anti-NY-ESO-1 directed T cells.

## Consent

Written informed consent was obtained from the patient for publication of this abstract and any accompanying images. A copy of the written consent is available for review by the Editor of this journal.

